# Toward a Framework for Islamic Psychology and Psychotherapy: An Islamic Model of the Soul

**DOI:** 10.1007/s10943-018-0651-x

**Published:** 2018-06-15

**Authors:** Abdallah Rothman, Adrian Coyle

**Affiliations:** Department of Psychology, Kingston University London, Penrhyn Road, Kingston-upon-Thames, KT1 2EE UK

**Keywords:** Islamic psychology, Islamic psychotherapy, Self, Soul

## Abstract

A uniquely Islamic theoretical framework for an Islamic psychology has yet to be established. To do so requires that we understand how human beings are conceptualized within the cosmology that characterizes the Islamic tradition. This paper presents a model of the soul from within an Islamic paradigm, generated through a grounded theory analysis of interviews with 18 key informants with relevant academic or religious expertise. The model elaborates aspects of a mechanism for the development of the soul that constitutes a potential foundation for an Islamic theory of human psychology and has particular relevance for Islamic approaches to psychotherapy.

## Introduction

Over the past few decades, the question of how best to define and realize the integration of Islam and psychology has gained much attention, culminating in what Kaplick and Skinner (2017) call an “Islam and psychology movement.” Despite the fact that in 1979 Malik Badri cautioned Muslim psychologists against adopting Western theories and called for an Islamic paradigm of psychology, the majority of research efforts within this movement over the past 10 years have focused on cultural or religious adaptations of Western models (Haque et al. 2016). A uniquely Islamic theoretical framework for an Islamic psychology has yet to be established. To do so requires that we understand at a foundational level how human beings are conceptualized within the cosmology that characterizes the Islamic tradition.

The understanding of human nature and the relative conception of structural aspects that make up the human psyche or “soul” determine much of how we make sense of behavior and motivation and are fundamental to the philosophical underpinnings of theoretical approaches to psychology and psychotherapy (Coon and Mitterer 2012). It is inappropriate to adopt the theoretical orientations that underpin contemporary Western psychology because most of these have been influenced by concepts of human nature other than those derived from Islam (Delaney and DiClemente 2005). Defining an Islamic psychology in an Islamically meaningful way would be impossible without first constructing a conceptual framework or model of human nature with Islamic integrity (Muhammad 1996). This would constitute a foundation for various forms of applied psychology such as Islamic psychotherapies. This paper engages with that task by presenting and analyzing the input of scholars from diverse disciplines of Islamic thought in the form of qualitative interview data and, through this analysis, developing an Islamic model of the soul.

Among the handful of studies that have attempted to tackle the ambitious project of developing a uniquely Islamic psychological framework, the work of Keshavarzi and Haque (2013) is important as it articulates many of the necessary considerations in this endeavor. They discuss a broad range of considerations, including cultural attitudes to and religious perspectives on mental health, but they focus on the four aspects of the soul (the *nafs* or lower self, the *qalb* or heart, the *aql* or intellect, and the *ruh* or spirit) that were identified/elaborated in the twelfth century by al-Ghazali (2015) and have been referenced by many authors in the ‘Islam and psychology’ movement (for example, Abu-Raiya 2012; Haque 2004; Skinner 2010). Keshavarzi and Haque (2013) outline how these four aspects can provide a framework for interventions, with each representing a “level” of the soul as a relative target point for intervening according to assessment and diagnosis. Prior to that, Skinner (2010) had alluded to a similar conception of assessment and treatment using these aspects as a guide.

Abu-Raiya (2012) presented a “systematic Qur’anic theory of personality” which also outlines a framework of the psychological nature of the human being around these four aspects but adds the three developmental stages as found in the Qur’an: *nafs al ammarah bil su* (soul that inclines to evil), *nafs al lawwama* (self-reproaching soul), and *nafs al mutmainah* (soul at rest). The inclusion of these stages is significant in that they impart a dynamic element to the framework and indicate a natural progression of the soul toward growth. The same applies to Briki and Amara’s (2018) “perspective of Islamic self” that is built on Ibn al Qayyim’s ideas (Haque 2004) and draws from a “dynamical social psychology,” whereas Abu-Raiya (2012) draws substantial comparisons with Freudian theory and uses it as a resource in his framework.

While there is a dearth of literature and research on the psyche, the self or the soul within the realm of Islam and psychology (Haque et al. 2016; Kaplick and Skinner 2017), these few papers have been useful in laying some groundwork and suggesting potential directions for further expansion of a theoretical framework for the domain. What all of these efforts have in common is that, although their concepts and principles were taken directly from sources within the Islamic tradition, the synthesis and understanding of these concepts were the product of only one or two individuals whose expertise is mainly in the field of psychology. In addressing the need to develop a comprehensive theory within an Islamic epistemological paradigm, Elmessiri (2006) asserted that the project must be a collaborative effort by a team of scholars who organize relevant thought and knowledge in a “creative attempt to apprehend the paradigms implicit in different Islamic texts and phenomena” (p. 68). This range of perspectives is necessary due to the variety of interpretations and definitions of Islam (Ahmed 2015; el-Zein 1977; Iqbal 2001), as well as the numerous branches of knowledge within the Islamic tradition that offer critical insight into and explanation of relevant teachings and concepts in regard to human psychology.

This paper presents a study that aimed to develop a data-grounded, consensual model of the soul from within an Islamic paradigm. The research question that underpinned the study was: what are the core principles and concepts regarding the conceptualization of the person from within an Islamic paradigm? It is hoped that, in later work, such a model could constitute a foundation for an Islamic theory of human psychology and Islamic approaches to psychotherapy.

## Method

### Participants

Participants were sought who had academic or religious expertise related to Islamic conceptions of human psychology and who could therefore act as “key informants,” sharing their own views and commenting from their informed positions on the views of others in their field (Gilchrist 1992). Forty individuals were ultimately identified and were invited to take part. Most were authors of books or articles relevant to the research question but others were identified through personal recommendations and “snowballing”. All were experts in their field and many were leading thinkers in topics related to Islamic psychology.

Eighteen people agreed to participate: 17 men and one woman who ranged in age from 39 to 89 years. Participants came from nine countries across four continents. The sample included practising Muslims, non-practising Muslims, and non-Muslims. Five participants were Islamic psychologists involved in research and/or clinical work; four were scholars in Islamic spirituality who were based in academic contexts; three were academic scholars in Islamic philosophy; three were non-academic Islamic religious scholars; three were traditional Islamic spirituality practitioners. Not all participants were recruited at the same time. Instead recruitment occurred progressively in a process referred to as “theoretical sampling” which characterized the research approach adopted in this study. This is explained below.

Except for the Islamic psychologists, many participants had little background in psychology and most were not previously familiar with the concept of Islamic psychology per se. While participants with an academic background provided historical and often interdisciplinary perspectives on Islamic thought, participants with a traditional religious background were able to provide context to practical applications of such knowledge.

### Data Generation

Data were generated through individual semi-structured interviews conducted by the first author. The interview schedule featured open-ended questions that were developed to elicit participants’ personal views and opinions on what might constitute an Islamic psychological understanding of the human being, based on their relevant knowledge and study of related fields. The topic of an Islamic conception of human psychology was introduced, and the participants’ personal stance and ideas were explored without specific direction from the interviewer: for example, “What are the key principles and concepts that might characterize an Islamic conception of psychology?” The interview schedule then focused on the particular principles that the participants identified as central, such as “How do virtues and vices come into play in the process of purification of the soul?”

When a favorable ethical opinion for the study had been obtained from the researchers’ university context, the field work began with piloting to test the schedule. Fifteen interviews were then conducted in person and three by video conference. The interviews lasted on average for 60 min but varied from 30 to 90 min. Each interview was digitally recorded and subsequently transcribed verbatim.

### Analytic Procedure

Transcripts were analyzed using the qualitative analytic procedure known as constructivist grounded theory, formulated by Charmaz (2014). This builds upon the original version of grounded theory developed by Glaser and Strauss (1967) who advocated “developing theories from research grounded in data rather than deducing testable hypotheses from existing theories” (p. 4). The approach enables new theoretical insights to be developed directly from a systematic interrogation of data and is particularly useful in areas where existing theory is incomplete, inappropriate or entirely absent (Charmaz 2014; Payne 2016). The constructivist approach to grounded theory methodology takes a relativist position, acknowledging the constructed nature of human experience and the multiplicity of possible standpoints of both participants and researchers. It views their input and the resulting analysis as part of a collaborative process of constructing and developing theoretical insights.

In accordance with the grounded theory approach, data generation and data analysis proceeded alongside each other. Data were engaged with through an initial process of “open coding” which involves identifying and labeling units of transcript text, for example, a word, phrase, sentence or larger section of text (Payne 2016). As these labels or “codes” were developed, they were compared to other codes, and (possible) relationships were recorded in process notes called “memos.” Analytic observations and conceptual connections gave rise to the construction of “theoretical categories,” which represent thematic concepts that organize the data relative to the developing theory or model (Charmaz 2014). During this process, sampling continued by recruiting new participants who could further elucidate emerging theoretical categories, an approach called “theoretical sampling” which is characteristic of grounded theory methodology (Glaser and Strauss 1967). The idea here is that, as theory is developed from the data, the question of where and with whom to sample next is answered by considering what aspects of the emergent theory or model might benefit from further clarification and elaboration.

The initial set of potential theoretical categories was then refined/developed through a process of “axial coding” (Charmaz 2014; Payne 2016). During axial coding, possible relationships between categories are noted, hypothesized and tested against data obtained in ongoing theoretical sampling until saturation is reached. Payne (2016) defines saturation as the gathering of further examples of meaningful units from the transcripts until no new instances of a particular category emerge. In this study, saturation was reached just before the eighteenth participant’s transcript was analyzed, as data collected from interviews at this point were only producing recurring codes and categories. The emergent model of an Islamic psychological conception of the soul was then re-“grounded” by going back to the data and validating it against actual text (Payne 2016). In the data excerpts that are used to illustrate the model in the next section, pseudonyms have been assigned to participants and their status is indicated when their pseudonym is first mentioned; dots indicate pauses in speech and empty square brackets indicate where material has been edited/excised.

## Results

**Fig. 1:**
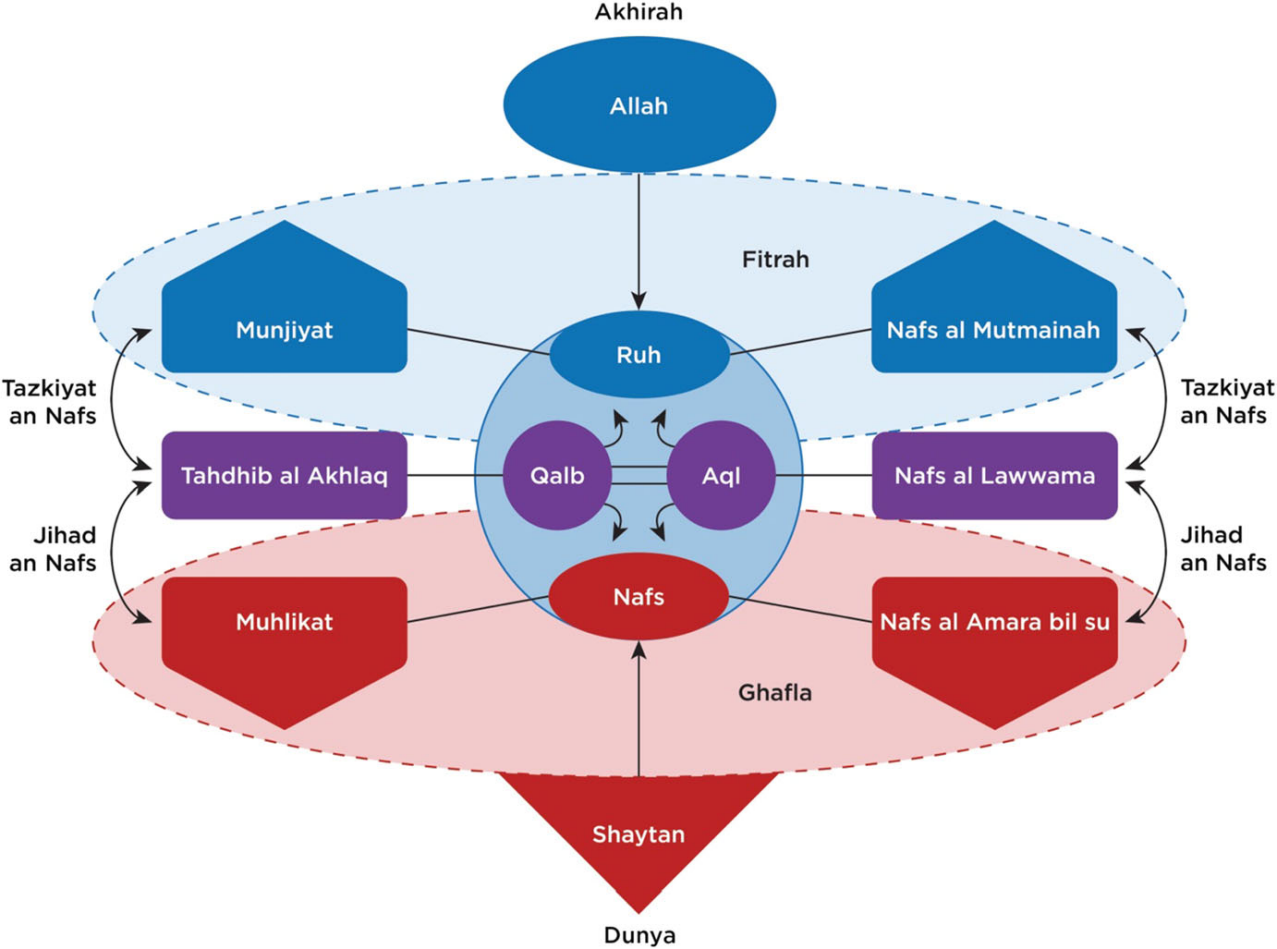
An Islamic model of the soul

The analysis produced eight theoretical categories relevant to the research questions. These categories and the relationships discerned between them constituted a coherent model of the soul, which is presented diagrammatically in Fig. 1 later in this paper. The concepts that make up the categories reflect the participants’ reliance on *tafsir* (exegesis) of the Qur’an and hadith in their interview responses, drawing upon writings of both modern and early scholars of Islamic theology and philosophy.

Although diverse insights were offered from different branches of knowledge and variations in interpretation were apparent, there was consensus about the distinct foundational elements of an Islamic conception of the soul. While all eight of the categories were noteworthy, four reflect more foundational aspects of the emergent model, namely “Nature of the soul,” “Structure of the soul,” “Stages of the soul,” and “Development of the soul” (see Table 1). Due to space constraints, we will elaborate in detail the category entitled “Development of the soul” as it has greatest relevance to applications of an Islamic psychology, particularly in Islamic psychotherapies. However, this category builds upon the other three foundational categories and so we shall first outline them and illustrate them sparingly from the data set.

**Table 1:** Theoretical categories and subcategories

Main categories	Subcategories
Nature of the soul	Concept of *fitrah*
	*Fitrah* exists underneath the projected self
	Being out of alignment with *fitrah*
	*Dunya* as distraction
	*Fitrah* as internal compass—realignment
Structure of the soul	Distinct features of the soul
	Soul as whole—integrated nature of the soul
	*Nafs* (lower self)
	*Qalb* (heart)
	*Aql* (intellect)
	*Ruh* (spirit)
Stages of the soul	Changing nature/fluctuation of the *nafs*
	*Nafs al amara bil su*
	*Nafs al lawwama*
	*Nafs al mutmainah*
Development of the soul	The human project of development
	*Tazkiyat an nafs* (purification of the soul)
	*Jihad an nafs* (struggle of the soul)
	*Tahdhib al akhlaq* (refinement of character)
	Need for moral reform
	*Muhlikat* and *munjiyat* (vices and virtues)

### Nature of the Soul

All 18 participants identified the concept of *fitrah*, defined as human nature or natural disposition, as being central to the conceptualization of an Islamic psychology. The consensus among participants was that *fitrah* posits that all human beings are born with the same sound nature, which most agreed is pure and which comes from and has a direct link to God. Yaqoub (a non-academic Islamic religious scholar) explained “the *fitrah* is an allusion to the original imprint of this *tawhid* [divine unity].” Participants reported that after conception a process of corruption begins, as the person makes their way through the trials of the *dunya* (temporal world) which begins to distance them from and cover over their pure nature.

Many participants related that having knowledge of God is a fundamental feature of the nature of the human soul from an Islamic perspective. John (an academic scholar of Islamic spirituality) explained that as a result of the *fitrah* “there is a deeper part of ourselves that knows God and we are naturally drawn back to that knowledge,” or as Abdelsalam (a non-academic Islamic religious scholar) said, “we have this primordial disposition to want to know Him… and that’s how we are hardwired.” In other words, the idea is that we may be out of touch with our knowledge of and witnessing of God, but we always have the ability to get back to it. Participants seemed to agree that the primary reason for most psychological problems is a misalignment with the *fitrah*. Yahya (an Islamic philosophy scholar) asserted “your psychological state has to be harmonious, your psyche, your mind … has to be harmonious with your *fitrah.*”

### Structure of the Soul

Many participants frequently used the word ‘self’ in reference to the Islamic concept of *nafs*. However, Mustafa (an Islamic psychologist) offered an important distinction that “the term ‘self’ as used in modern psychology is an immaterial complex psychological concept but *nafs* in Islam refers to a real spiritual being inhabiting our physical body. It is the soul.” In discussing the structure of the soul, most participants referenced the four main aspects (*nafs*, *qalb*, *aql*, and *ruh*) presented in al-Ghazali’s twelfth century work the *ihya ilumidin* (*The Revival of the Religious Sciences*, 2015) which are used to describe different functions or qualities of the one integral soul. Participants explained that every human being has forces of good and evil fighting in the battleground of his or her soul and, although one integrated whole, the distinction between these aspects helps us understand different functions that play a part in this struggle.

In this context, the term *nafs* is used to refer to the lower self, similar to the ego, in that it is the part of the soul that inclines toward the *dunya* through desires, distracting a person from Allah and opening them to the influence of *shaytan* (the devil). The *qalb* was explained by participants to be the spiritual center of the human being and a pivotal part of what determines the relative state of the soul. Participants described the *qalb* as having the ability to turn either toward the *dunya* and *shaytan* via the *nafs* or toward Allah via the higher aspect of the soul, and, as John and Cesar (academic scholars of Islamic spirituality) pointed out, the root of the Arabic word means “to turn.” A unique feature of the *qalb* that was reported is that it is the place where consciousness resides. This cognitive aspect of the *qalb*, participants indicated, is often referred to as the *aql* and was explained as the part of the *qalb* that “intellects,” with emphasis on the verb form of the Arabic word as used in the Qur’an. The *ruh* was explained as the part of the soul where God’s imprint resides within the *fitrah*, and it was reported to be unchanging and pure. Participants saw the *ruh* as being unique to an Islamic psychological conception of the soul in that it functions as a direct access point to God, where the human being can potentially receive divine knowledge, guidance, and healing.

### Stages of the Soul

Whereas participants specified that the *ruh* is an aspect of the soul that does not change, the other aspects of the soul are always in a state of flux, causing the soul to go through stages of development. Yahya specified that this changing nature of the *nafs* is why we need to be diligent in keeping the *qalb*, *aql* and *nafs* aligned and on the right path by exerting effort in the struggle of the soul. It was understood that our relative engagement with and success in that struggle determines the state of our soul and which of the stages we are in at any given time. While there were discrepancies as to how many stages exist, with five or even seven referenced, most participants agreed that the three main stages, as found in the Qur’an, are: *nafs al ammarah* (soul that inclines to evil), *nafs al lawwama* (self-reproaching soul), and *nafs al mutmainah* (soul at rest).

*Nafs al ammarah* was explained as a state in which a person is not exerting concerted effort in controlling the *nafs*, and essentially allowing the lower self to run wild. Yaqoub specified that this stage/state is not necessarily characterized by evilness per se but that it is an “evilness premised on the state of individuation,” where the person is anchored in selfishness rather than in awareness of God and they are in a state of *ghafla* (forgetfulness of God). *Nafs al lawwama* was described by participants as the stage when most of the work on the soul is being done. It was conceived as the place of the battleground where a person strives to resist the downward pull toward *nafs*, *dunya*, and *shaytan* and reach toward the *ruh*, *akhirah* (afterlife), and Allah through diligence and self-awareness. It was reported that people rarely cross into the final stage, *nafs al mutmainah*, and that even those who do are liable to fall back down to the lowest stage if lacking discipline. Therefore, *nafs al mutmainah* was talked about as the ideal to strive for more than something that is really expected to be achieved. Still it was conceived as having great importance as it posits the trajectory toward development that all participants seemed to feel was an integral aspect of the Islamic conception of the soul.

### Development of the Soul

#### The Human Project of Development

In the conception of the structure of the soul and understanding the makeup of the human being and human nature from a psychological point of view, it was almost impossible for most of the participants not to equate this knowledge directly with the process of purification of the soul. Several participants pointed out that the Islamic paradigm of understanding the human being views the purpose of human life as an opportunity to purify the soul and many described it as a project of development: to uncover the *fitrah* inside by purifying the *nafs*. Thus, as both Rahim (an Islamic psychologist) and Abdelsalam specifically pointed out, any conception of an Islamic psychology would necessarily involve this purification process, and it would be absurd to envisage a study of psychology without it. Rahim said, “It’s a more complete existence. It’s not focused on just getting people back into the capitalist system for instance, and just defining human functioning as being productive in a material sense,” pointing out that a Western approach to psychology would seldom include the state of the soul in a treatment plan.

Another idea that Abdelsalam presented was that an Islamic perspective would not have any use for the study of the soul without a direct link to treatment. He said:

“So it is not just the study of psychology but the process of cleansing, the process of cleaning, making the mirror shine. You know the *nafs* is like a mirror and it is turbid and it needs to reflect the light of the divine, you know, and it’s turbid, full of dirt. So these are all benefit for the study, so it is not just like studying the *nafs* for the sake of the *nafs*.”

He went on to draw the analogy of the study of anatomy versus the treatment of illness in the body, and that an Islamic perspective of psychology is inextricably linked to the process of cleansing the soul. He then pointed out that while the terms *ilm an nafs* (knowledge of the soul) or *fiqh an nafs* (deep knowledge of the soul)—as Yahya said al-Ghazali used—are more about the study of the structure of the soul, *tazkiyat an nafs* (purification of the soul) was traditionally viewed as a practical way of living and is more about putting that knowledge into application, or the process of shining the mirror.

#### *Tazkiyat an nafs* (Purification of the Soul)

Almost all participants used this metaphor of shining a mirror in reference to doing work on the soul to clean the heart and uncover the *fitrah*. The usefulness of this metaphor seemed to be that it exemplifies the idea that the purity or light that one is attempting to shine is not the person’s own, but that their soul can be a reflection of the divine light if cleaned and if the crust is removed that accumulates from the illusion of separation resulting from life in the *dunya*. In reference to this Cesar said, “the *nafs* has an ability to kind of cover up the *ruh* so that really with the *ruh* it’s about polishing and clearing and letting the light, letting the *ruh* itself shine.”

One term commonly used in reference to this cleaning process is *tazkiyat an nafs*, a deep process of inner work to purify and perfect the soul to allow it to shine in its highest state, the essence of that higher state being the *ruh*. Both Shaykh Abdalbarr and Shaykh Aziz (traditional Islamic spirituality practitioners) described this process as a higher level of purification and attributed it to a person perfecting their intentions and actions by doing things in addition to minimum requirements, such as extra worship. This can be seen as a more advanced form of “polishing the mirror” work. However, according to several participants, this is a higher spiritual state than most people will achieve in life, closer to the stage of *nafs al mutmainah*. In reference to the writings of the ninth century Islamic philosopher Harith al Muhasibi, who was influential in the development of an Islamic conception of psychology, Gareth (an academic scholar of Islamic spirituality) explained that:

“He focuses on the undeveloped form of the soul. So, in other words, you know, the whole idea of purification takes one to the highest level – the *nafs al mutmainah* or the tranquil, serene soul. But he doesn’t really focus on that. He focuses on what your soul is like now, in its kind of general state – what most people experience. That’s his main focus, I think that’s important.”

Several participants talked about that which is of relevance to the majority of people as they struggle to simply chip away at the crust covering the mirror rather than polishing an already mostly uncovered reflection. This more fundamental process was commonly referred to as *jihad an nafs* (struggle of the soul) and, although *tazkiyat an nafs* could be included in this term, *jihad an nafs* was conceived to be more generally referring to work on the soul at any level.

#### *Jihad an nafs* (Struggle of the Soul)

Thus, the main focus for most of the participants in asking them to conceive of an Islamic paradigm of the person in relation to psychology was that it primarily entails struggling against the powerful influence of the *nafs* in the process of trying to come back into alignment with *fitrah*. It was reported that this is essentially what is at the heart of the *deen* (religion) of Islam and what much of the commentary on the Qur’an elaborates. As Yahya pointed out, in reference to the scholars who wrote those Qur’anic commentaries, “It’s the *mujahada*, the struggle over the *nafs*, it’s back to psychology. It’s just that they don’t call it psychology [] We’re calling it psychology.” He then went on to say:

“Islamic psychology is to do with those *nafs*, the *jihad* – the *mujahada*, the struggle over the *nafs*. Because when we struggle… because the *nafs* is this… is sort of influenced by certain contingent happenings from the environment, from our instincts or from environmental influences that cause us to deviate from *fitrah*.”

This struggle involves looking at the state of the soul and being self-reflective. Therefore, participants explained that it generally happens within the stage of *nafs al lawwama*, when the person is able to have self-reproach. In reflecting on the self, as John said, “It’s thought that one would find out where one’s weaknesses lie. And one has to deal with them.” The *jihad* (struggle) then is not simply about fighting against the *nafs*; rather it involves a constructive response to the discovery of faults.

It was explained by many participants that the act of dealing with these weaknesses, lower impulses or bad character traits involves training the *nafs* and following the guidance from the Islamic tradition as a map to the desired outcome. Hamit (an Islamic psychologist) said, “We believe foundationally that there’s a training process, that the training process entails that one try to bring one’s *nafs* or self in conformity with the Islamic tradition.” Yahya said, “and that’s why we need Qur’an and Sunnah [prophetic tradition]—to guide us on to that path, to bring us back to our state of *fitrah*.” Thus, from the viewpoint of an Islamic paradigm of psychology, the religious obligations and advice from the Qur’an along with the example of the Prophet Muhammed were represented by all participants as the treatment for the *nafs* in the process of reform. Whereas this guidance is in the form of a holistic, all-encompassing path of life and one that is embarked upon over the course of a lifetime, it was not thought of as an easy fix. In explaining this process of conforming to the guidance from the Islamic tradition, Hamit said, “That’s not an easy process to begin and at the beginning that’s not gonna feel very natural, and it’s normal to allow for that discomfort… towards the discipline of formulating the *nafs*.”

#### *Tahdhib al akhlaq* (Refinement of Character)

##### Need for moral reform

While there may be fairly consensual ideas of well-being and ideal functioning, Western psychology does not necessarily have one universal set of moral guidelines (however that is understood) to hold people accountable to or encourage alignment with, whereas an Islamic psychology necessarily involves the moral framework and guidance set out by the Qur’an and Sunnah as the benchmark for human ideals. In reference to the Western model, Hamit said, “It’s relativistic…there’s all ‘small ts,’ there’s no ‘big T’. It’s whatever you really feel inclined towards and that we trust that human inclination.” The “t” that Hamit is referring to here is “truth.” In other words, from this paradigm of secular generality that Hamit is invoking, the proposition is that everyone’s own truth (small t) is potentially equally valid, with little to no universal or objective truth (big T) that people are expected to be held accountable to, outside of the general expectation to protect the well-being of self and others. Many participants talked about the need for moral reform, for a person to work on improving their character, as an integral part of what must happen in the stage of *nafs al lawwama*, on the path of aligning with *fitrah* and striving toward the stage of *nafs al mutmainah*.

In talking about this need for moral reform in the Islamic conception of human psychology, Tarkki (an Islamic philosophy scholar) referenced the importance of some of the early classical scholars of Islamic philosophy and their “treatises in moral philosophy, with people like Yahya ibn Adi or Miskaweh, each of whom produced a work called the reformation of morals or the…sort of the improvement of character, *tahdhib al akhlaq.*” *Tahdhib al Akhlaq* literally means the reformation or refinement of character and, similar to *tazkiyat an nafs* and *jihad an nafs*, involves working on the *nafs*. The distinction with *tahdhib al akhlaq* however is that it is specifically about redirecting blameworthy character traits and adopting praiseworthy ones. This involves, again, redirecting away from what the *nafs* wants, with the assumption that those desires are generally not what is best for the person and may lie at the heart of psychological distress or disorder. As Cesar put it, “With the uncontrollable, insatiable, you know, desires the human beings have can lead to tremendous moral problems.” Participants specified that these inclinations are not seen as psychological deficiencies in the person but as normal characteristics of the uncontrolled *nafs*. John said, “It’s understanding these as not just fissures of the psyche, but inclinations of the *nafs* and treating them on that level as inclinations of the *nafs.*” Similarly, Yaqoub pointed out that this view does not cause the person to identify with a diagnosis and perceive it to be static but instead to view it as a passing state that has a known treatment.

##### *Muhlikat* and *munjiyat* (Vices and virtues)

The Greek philosophy tradition of virtue ethics, as explored by the likes of Plato and Aristotle, and the connected field of moral psychology were influential in the development of discourse on human psychology by classical Islamic scholars (Iqbal 2013). Tarkki argued that, “Al-Ghazali is far more focused than any other author I know up to that point…in trying to figure out what the systematic bases for our various shortcomings, psychological shortcomings, would be… so the moral psychology aspect.” Many of the participants recognized the significant contribution that Islamic scholars like al-Ghazali offered to the field of moral psychology, particularly those who had an academic orientation to the study of Islamic philosophy and spirituality and the history of such thought. Gareth pointed out that while these scholars may have been influenced by Greek philosophy, they had a different orientation to the application and purpose of such knowledge. He said, “They’re really looking at it from the paradigm of what is the method to get you out of these character traits and get into the character traits that you should have that will make you purified.” So, vice and virtue, in the Islamic context, become specifically about illness and treatment in the process of purifying the soul.

Almost all of the participants who spoke in detail about the process of *tahdhib al akhlaq* identified al-Ghazali’s contribution as being most significant in that he developed a systematic framework for the treatments of these illnesses of the soul in his discussions on the *muhlikat* (vices) and *munjiyat* (virtues). In describing al-Ghazali’s system Abdelsalam said, “The *munjiyat* are the things that help you to get to your goal but the *muhlikat* are the things that keep you away from your goal.” Often the cure for a certain vice is its opposite in the form of a virtue, as Abdelsalam went on to say, “because to rule with justice you need to get rid of *zulm* [injustice], which is the opposite to justice.” Enas (an Islamic psychologist) pointed out that not only did al-Ghazali give an exhaustive list of illnesses, or *muhlikat*, and their treatments, or *munjiyat,* but he also outlined a program for how to go about treating each one, found particularly in book three of his *ihya ilumidin.*

Participants described the *muhlikat* as natural tendencies within the *nafs* of the human being which pull a person toward the lower part of the self and a downward trajectory in the realm of *dunya* and the forces of *shaytan*. Yahya said, “At the psychological level we have this tendency towards greed, towards power, towards lust, and so on.” Often many of these destructive character traits were conceived of as appetites of the *nafs*. Tarkki said:

“Appetites and the spirited part of our soul in various ways have to do with our more selfish impulses…but at the same time they…don’t allow for an alignment of the way we live our lives with the sort of larger order of the universe or of reality and of course that’s detrimental to us ourselves.”

These appetites were understood as distractions from alignment with our *fitrah* and as needing to be systematically controlled and disciplined. In discussing al-Ghazali’s writing on the *muhlikat*, Kyle (an Islamic philosophy scholar) said that “He talks about breaking the appetite of hunger…pursuing a strategy to try to break hunger’s hold on us.”

In addition to base bodily desires such as hunger, participants noted that al-Ghazali gives a great deal of attention to vices of the heart, where the *nafs* influences the *qalb* and infect it with things like greed, envy, or anger, a trait that gets an entire chapter in the *ihya illumidin* due to its apparent centrality in the downfall of human character. Also given a great deal of focus is the general tendency for human beings to think highly of themselves and be selfish. Tarkki described the danger in this:

“The desire for status and or whether it’s sort of superiority over our neighbors or just feeling good about ourselves or sort of papering over our shortcomings or failure to want to own up to them, and so on, that that leads to us even to sort of aim for the wrong kinds of things in life and to be reticent about going to any kind of hard work in genuinely improving ourselves.”

The *munjiyat* were described by participants as the Godly qualities that are a part of our birthright within our *fitrah*, and connected to the *ruh* aspect of the soul. They noted that when we adopt these character traits as treatments for the illnesses of our *nafs*, we get closer to embodying that innate Godly nature within and, as Abdelsalam pointed out, this is where we can access our higher purpose as human beings as *khalifahtullah* (vice-regent of God). In referring to what is meant by the biblical as well as Qur’anic saying that humans were created in God’s form, Abdelsalam explained that “He has made us such that we can rule over ourselves and over … you know … with the same justice and wisdom.” Thus, by demonstrating these qualities sincerely and authentically, the notion is that we elevate our status and move upward on the trajectory toward *nafs al mutmainah*, this being the focus of human existence in the *dunya* and therefore the focus of an Islamic perspective of the psychology of the person.

## An Islamic Model of the Soul

The model presented in this paper and illustrated in Fig. 1 was developed from the participants’ consensual understanding of the nature, structure and development of the soul as it relates to human psychology from the Islamic tradition. The theoretical categories are a synthesis of different sources and different strains of Islamic scholarship and spiritual-religious knowledge. However, the research interest in developing a model that, in later work, could constitute a foundation for Islamic approaches to psychotherapy, as well as for an Islamic theory of human psychology, may have oriented participants (and the analysis) more to some sources (for example, al-Ghazali) than others. Hence the resultant Islamic model of the soul is best thought of as a contribution to “a” theory of Islamic psychology, with a recognition that there could conceivably be multiple versions of Islamic psychology or Islamic psychologies.

Drawing together the elements that we have elaborated, according to this model, the human soul has an innately pure and good nature, *fitrah*, that comes from and is connected to God but that becomes covered over and forgotten as a natural part of life in the *dunya*. Throughout its life in the *dunya*, within the soul there exists a dynamic interplay of conflicting forces that affect the psychological state of the person and determine relative levels of alignment or misalignment with *fitrah.* This process is represented by the purple elements in the middle of the model in Fig. 1.

The *qalb*, which is the spiritual center of the person, and where the faculty of intellect is located as the *aql*, has the potential to turn in either of two directions which shapes the relative, transient outcome of this conflict. It can turn toward the lower impulses of the *nafs* and become further misaligned with *fitrah* by the influences of the *dunya* and *shaytan*, resulting in increased negative characteristics of the *muhlikat* and a state of *ghafla*. This process is represented by the red elements toward the bottom half of the model in Fig. 1, from the *nafs* downward. Or it can turn toward the higher, Godly nature of the *ruh* with the remembrance of *Allah* and the *akhirah* (afterlife), resulting in increased positive characteristics of the *munjiyat*, and come more in alignment with the soul’s state of *fitrah.* This process is represented by the blue elements toward the top half of the model in Fig. 1, from the ruh upwards.

The relative state of the soul in relation to either of these two poles at any one time is articulated in three distinct stages of the soul’s development throughout life in the *dunya*, namely: *nafs al ammarah bil su*, *nafs al lawwama*, and *nafs al mutmainah*. The model posits that the soul has an inherent inclination toward growth and an upward trajectory in relation to this model, due to its primordial nature of knowing God, and that the Islamic tradition, as guided by the Qur’an and Sunnah, encourages and maps out a path for the human being to pursue this trajectory. This is demonstrated in the description of processes along the path that act as mechanisms for exerting effort in the dynamic interplay within the soul as it struggles between the two opposing forces, namely *jihad an nafs*, *tahdhib al akhlaq*, and *tazkiyat an nafs*.

These findings reflect and support those reported by Abu-Raiya (2012) and Keshavarzi and Haque (2013), particularly in the centrality of the elements of the soul (*nafs*, *qalb*, *aql*, *ruh*). However, the specific dynamics of how these aspects interrelate and interact with each other were under-developed in Keshavarzi and Haque’s (2013) work. Those dynamics are a key feature of the model developed in the present study which, in its consistently Islamic grounding, diverges more significantly from the work of Abu-Raiya (2012). His Qur’anic theory of personality follows some of the a priori assumptions of Freudian theory and “holds a largely negative view of human nature” (p. 231), which more closely resembles a Christian paradigm of human nature (Niebuhr 2004).

In addition to a more positive view of human nature in the sense of the soul being innately pure, the Islamic model of the soul presented in this study possesses several features that distinguish it from most secular Western models of human nature. The notion that the spiritual center of the human being is the heart is a significant distinction, together with the contention that the intellect and consciousness are located in this heart center rather than in the mind, as most psychological theories posit. Furthermore, the idea that this center of consciousness within the human being is inherently connected and can be consciously connected to a primordial, divine consciousness is absent from Western, secular theories of human nature. The concept of the *ruh* as a point of access within the person which can directly receive guidance and/or healing from God and the utilization or lack of acknowledgement of this aspect within psychotherapy could have a significant impact on therapeutic guidance and treatment goals for Muslim clients. These, along with other crucial conceptual differences highlighted in the findings, suggest the need for more than just Islamically adapted or integrated approaches to psychotherapy. They substantiate the necessity of a unique framework grounded in an Islamic paradigm, as represented by this model and any theory of Islamic psychology of which it is a foundational constituent. Building upon this model, the three aspects identified here that collectively form a mechanism for the development of the soul (*tazkiyat an nafs*, *tahdhib al akhlaq*, and *jihad an nafs*) have particular relevance to application in psychotherapy and implications for further research in the development of indigenous approaches. A useful next step in deepening the model and advancing those endeavours would be to engage analytically with relevant classical and modern Islamic texts, including those invoked by participants in the present study, and with the views and experiences of practitioners of versions of Islamic psychotherapy and of psychotherapy with Muslim clients. The authors are presently engaged in this work, which extends and builds upon the foundations offered in this article, and look forward to sharing their findings in due course.
